# Microporous PdCuB nanotag-based electrochemical aptasensor with Au@CuCl_2_ nanowires interface for ultrasensitive detection of PD-L1-positive exosomes in the serum of lung cancer patients

**DOI:** 10.1186/s12951-023-01845-y

**Published:** 2023-03-11

**Authors:** Luyue Chang, Haiping Wu, Rui Chen, Xiaoqing Sun, Yi Yang, Changwu Huang, Shijia Ding, Changjin Liu, Wei Cheng

**Affiliations:** 1 The Center for Clinical Molecular Medical Detection, The First Affiliated Hospital of Chongqing Medical University, Chongqing, 400016 China; 2 Department of Laboratory Medicine, The Fifth People’s Hospital of Chongqing, Chongqing, 400062 China; 3 Key Laboratory of Clinical Laboratory Diagnostics (Ministry of Education), College of Laboratory Medicine, Chongqing Medical University, Chongqing, 400016 China

**Keywords:** PD-L1^+^ exosome, Electrochemical aptasensor, Microporous PdCuB nanotag, Au@CuCl_2_ nanowires, Non-small cell lung cancer

## Abstract

Programmed cell death ligand 1 protein-positive (PD-L1^+^) exosomes have been found to be a potential biomarker for the diagnosis of non-small cell lung cancer (NSCLC). However, the development of highly sensitive detection technique for PD-L1^+^ exosomes is still a challenge in clinical applications. Herein, a sandwich electrochemical aptasensor based on ternary metal-metalloid palladium-copper-boron alloy microporous nanospheres (PdCuB MNs) and Au@CuCl_2_ nanowires (NWs) was designed for the detection of PD-L1^+^ exosomes. The excellent peroxidase-like catalytic activity of PdCuB MNs and the high conductivity of Au@CuCl_2_ NWs endow the fabricated aptasensor with intense electrochemical signal, thus enabling the detection of low abundance exosomes. The analytical results revealed that the aptasensor maintained favorable linearity over a wide concentration range of 6 orders of magnitude and reached a low detection limit of 36 particles/mL. The aptasensor is successfully applied to the analysis of complex serum samples and achieves the accurate identification of clinical NSCLC patients. Overall, the developed electrochemical aptasensor provides a powerful tool for early diagnosis of NSCLC.

**Supplementary Information:**

The online version contains supplementary material available at 10.1186/s12951-023-01845-y.

## Introduction

Non-small cell lung cancer (NSCLC) is the most lethal of major cancer types, accounting for approximately 85% of all lung cancer patients [1]. The median overall survival and 5 year survival rates of NSCLC patients are very low [2]. Thus, early, sensitive and selective diagnosis of NSCLC is beneficial to improve the treatment efficacy and survival rate of these NSCLC patients. Currently, tissue biopsy is regarded as the gold standard for NSCLC diagnosis, but inapplicable to early screening due to invasive sampling and complex technical requirements. Serum protein markers, such as cancer antigen 125 (CA125) and squamous cell carcinoma-associated antigen (SCC), are convenient to sample, but lack specificity for diagnosing NSCLC. There is no sensitive and specific technique available for early diagnosis of NSCLC so far. In recent years, studies have confirmed that the existence of tumor cell-derived exosomes in the peripheral circulation at the early stage of tumor development, which can change the pre-metastatic microenvironment and drive organ-specific metastasis [3, 4]. Particularly, exosomes with high surface expression of programmed cell death ligand 1 (PD-L1) have been found in peripheral blood of NSCLC patients, suggesting that PD-L1-positive (PD-L1^+^) exosomes are potential serum markers for early and accurate diagnosis of NSCLC, which is clinically important for improving the survival rate of NSCLC patients [5, 6]. A variety of techniques have been employed for the quantitative determination of exosomes, such as enzyme-linked immunosorbent assay (ELISA), surface plasmon resonance imaging, electrochemical technology, western blotting, and flow cytometry [5, 7, 8]. Among them, electrochemical technique is considered as an attractive approach owing to the advantages of easy integration, high sensitivity and simplicity. For example, a facile sandwich electrochemical strategy has been developed for highly specific biomarker detection by integrating the naturally strong affinity of inducer and receptor [9]. Conventional electrochemical biosensors are usually powered by biocatalytic reaction, which suffer from unstable and limited-lifetime output signal [10]. For this reason, non-enzymatic tags, especially nanozymes, instead of enzymes, have been employed for constructing electrochemical biosensors with intense and long-lasting signals due to their properties of high stability, tunable size and composition, and ease of mass production and modification [11–13].

Microporous noble metal nanospheres are considered ideal nanotags for improving the catalytic performance of electrochemical sensors due to the accelerated transport of substances in three-dimensional interconnected microporous channels and altered interfacial electronic state of noble metals such as palladium (Pd) [14, 15]. Furthermore, noble metal-based alloys incorporating economic transition metals such as copper (Cu) as electrocatalysts for oxygen reduction reaction (ORR) are conducive to improving the catalytic activity [16]. Besides, the incorporation of metalloid elements such as boron (B) to construct multi-component alloys is an effective strategy to improve the electrocatalytic activity of the meta catalysts ascribing to the influence of multivalent electrons on the electronic structure of the metal [17, 18]. Based on the above report, designing ternary microporous PdCuB nanospheres (PdCuB MNs) and introducing them into sandwich electrochemical biosensors as signal tags are of great significance to improve the detection sensitivity. The strong conductivity of the electrode is also essential to highly sensitive electrochemical biosensor [19]. Copper chloride (CuCl_2_) nanowires (NWs) as an electrode modifier exhibits good electrical conductivity and high chemical stability [20]. As a classical conductive substance, gold (Au) exhibits high conductivity and excellent biocompatibility, has been widely used in electrode modification and biological analysis [21, 22]. Thus, the development of gold@copper chloride (Au@CuCl_2_) NWs modified electrode is beneficial to tether the aptamer (Apt.) and exploit sensitive electrochemical biosensor.

**Scheme 1:**
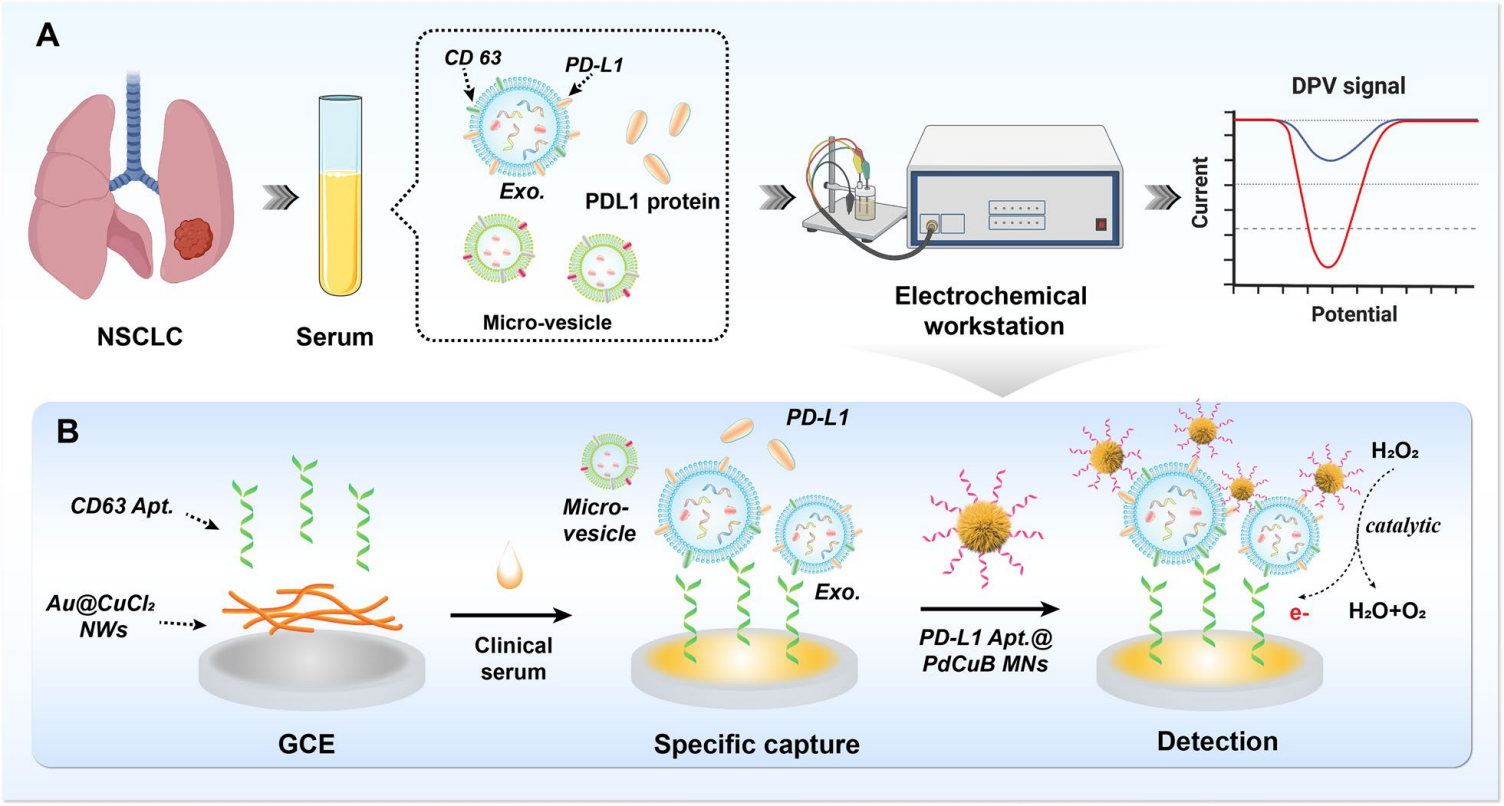
Illustration of the sandwich electrochemical aptasensor for PD-L1^+^ exosome analysis. **A** Procedure for electrochemical detection of PD-L1^+^ exosomes in clinical serum. **B** Assembly of electrochemical aptasensor and exosome detection process

In this work, we report the facile preparation of PdCuB MNs and Au@CuCl_2_ NWs, and demonstrate their huge application potential in sandwich electrochemical non-invasive sensor for highly sensitive detection of PD-L1^+^ exosomes (Scheme 1). As shown in Scheme 1, A micro-vesicles and exosomes are both extracellular vesicles formed by cellular outgrowth and secretion via the plasma membrane, the difference lies in the particle size and the extracellular protein. The CD63 on the surface of exosome membrane can be considered as the major marker to distinguish micro-vesicles from exosomes. Au@CuCl_2_ NWs are assembled into a substrate platform to improve the conductivity of electrode. The CD63 Apt. is tethered to the Au@CuCl_2_ NWs modified electrode for selectively capturing exosomes. In addition, the ternary PdCuB MNs with peroxidase-like activity are synthesized and modified by PD-L1 Apt. for enhanced electrochemical signals and the specific recognition of PD-L1^+^ exosomes. In the presence of PD-L1^+^ exosomes, the PD-L1 Apt. modified PdCuB MNs aggregate on the CD63 Apt. tethered Au@CuCl_2_ NW modified electrode, forming a sandwich structure on the surface of electrode. Furthermore, the electrochemical signal is generated with the addition of hydrogen peroxide (H_2_O_2_) due to the favorable peroxidase-like catalytic activity of ternary PdCuB MNs. The developed electrochemical inducers represent a promising strategy for exosome detection in early cancer screening.

## Experimental

### Reagents and materials

HPLC-purified DNA oligonucleotides were ordered from Sangon Biotech Co., Ltd. (Shanghai, China). All oligonucleotide sequences are listed in Additional file 1: Table S1. Dioctadecyldimethylammonium chloride (DODAC) and borane dimethylamine complex (DMAB) were obtained from Alfa Aesar (Ward Hill, USA). Hypophosphite (NaH_2_PO_2_·H_2_O) and ammonium fluoride (NH_4_F) were purchased from Sigma‒Aldrich (St Louis, USA). Palladium (II) chloride (PdCl_2_), cupric nitrate (Cu(NO_3_)_2_), anhydrous cupric chloride (CuCl_2_), boric acid (H_3_BO_3_), and thiourea were obtained from Adamas-beta (Shanghai, China). Anhydrous ethanol, hydrochloric acid (HCl), and ammonia solution (NH_3_·H_2_O) were provided by Chuandong Chemical Co., Ltd. (Chongqing, China). In this research, all reagents were at least analytical grade without further purification. Milli-Q ultrapure water (Millipore, ≥ 18 MΩ/cm) was used throughout.

### Apparatus

Electrochemical measurements were conducted using a conventional three-electrode system in a PC-controlled Donghua DH7000 electrochemical workstation, with a glassy carbon electrode (GCE) as the working electrode, a platinum wire as the counter electrode, and an Ag/AgCl electrode (saturated KCl solution) as the reference electrode. Transmission electron microscopy (TEM), high-resolution TEM, and energy dispersive spectrometer (EDS) mapping images were obtained on a JEOL JEM-2100F operating at 200 kV. X-ray photoelectron spectroscopy (XPS) data were recorded on a Thermo Scientific ESCALAB 250Xi spectrometer with Al Kα radiation. Scanning electron microscopy (SEM) images were collected using a Hitachi S-4800 operating at 3 kV. Nanoparticle tracking analysis (NTA) measurements were performed by using a Particle Metrix ZetaView PMX110 instrument.

### Cell culture, exosome preparation and characterization

Human non-small cell lung cancer cell lines (A549 cells) were cultured with 10% fetal bovine serum (containing 1% penicillin/streptomycin) at 37 °C in a humidified atmosphere containing 5% CO_2_. When the cell confluence reached 70%, the supernatant containing exosomes was collected and kept in the culture medium free of fetal bovine serum for 12 h. Subsequently, exosomes were separated from the culture medium via differential centrifugation. Briefly, the collected supernatant (150 mL) was centrifuged at 2000 × g for 30 min, and then continuously centrifuged at 10000 × g for 30 min to remove all the cell debris. Afterward, the supernatfant was subjected to membrane filtration (0.22 μm pore size), followed by centrifugation at 110000 × g for 70 min and centrifugal filtration (50 kDa) at 3000 × g for 15 min to concentrate the solution to 40 mL. The resulting solution was then treated by subsequent membrane filtration (0.22 μm pore size) and ultracentrifuged at 110000 × g for 90 min again to obtain PD-L1^+^ exosome. The obtained exosomes were stockpiled at − 20 °C for subsequent characterizations and experiments.

### Synthesis of PdCuB MNs

PdCuB MNs was prepared according to a previous report with some modifications [23]. Typically, 120 mg DODAC was dispersed into 40 mL deionized H_2_O in a 100 mL beaker, and the mixture was sonicated and stirred at 25 °C to obtain an albescent homogeneous suspension. Afterwards, 4 mL of NH_4_F solution (0.337 M), 4 mL of H_3_BO_3_ solution (0.101 M), 2.4 mL of H_2_PdCl_4_ solution (10 mM), and 0.8 mL of Cu(NO_3_)_2_ (10 mM) were added sequentially and the mixture was gently shaken for 10 min. Then, 2 mL NH_3_·H_2_O (10 wt%) was slowly added under stirring and the color of the mixed solution changed from reddish to light-white. The mixed solution was continuously stirred at 85 °C for 25 min. After 4.0 mL freshly prepared DMAB solution (0.1 M) was injected into the above solution, the solution immediately turned dark black, indicating the formation of PdCuB MNs. The products were obtained by centrifugation and washed with ethanol/H_2_O several times. Finally, the PdCuB MNs were weighed and dispersed in 4 mL with a final precipitation concentration of 2 mg/mL storage at 4 °C for further use.

### Synthesis of PD-L1 Apt.@PdCuB MNs nanotags

100 μL the as-prepared PdCuB MNs solution was added to 400 μL PD-L1 Apt. (1 mM) and incubated at room temperature overnight. Afterward, 50 μL aging buffer (pH 7.4; containing 10 mM PBS, 0.1% SDS and 0.3 M NaCl) was added, repeated 5 times at an interval of 1 h and then incubated for 12 h. The unconjugated Apt. were discarded by centrifugation and PD-L1 Apt.@PdCuB MNs nanotags were collected. The final product was dispersed in 1 mL PBS (10 mM, pH 7.4) and stored at 4 °C for further use in electrochemical analysis.

### Synthesis of Au@CuCl_2_ NWs

CuCl_2_ NWs were synthesized as a previously report with some modifications [20]. Specifically, 0.025 g CuCl_2_ and 0.025 g thiourea were dissolved in 20 mL ethanol and sonicated until the formation of a white suspension. The product was collected via centrifugation and washed twice with ethanol. The obtained CuCl_2_ NWs was dispersed in 20 mL anhydrous ethanol for further use. Then, 0.005 g AuCl_3_ was dissolved in 10 mL ethanol to prepare AuCl_3_ solution. Subsequently, the as-synthesized 20 mL CuCl_2_ NWs suspension was mixed with 5 mL AuCl_3_ solution and stirred for 30 min. After centrifugation and repetitive washing, Au@CuCl_2_ NWs were resuspended in 20 mL deionized water for characterization and electrochemical application.

### Fabrication of the electrochemical aptasensor

The bare GCE was routinely polished with an alumina (0.05 μm Al_2_O_3_) suspension. Afterward, the polished GCE was rinsed with ultrapure water in an ultrasonic bath and dried under nitrogen gas for further use. To fabricate the modified electrode, 10 µL of the Au@CuCl_2_ NWs suspension was drop-cast on a clean GCE surface and dried at 37 °C. Then, 10 μL CD63 Apt. (5 μM) was added and incubated with the modified GCE at 4 °C for 8 h. After each step, the modified electrode was washed with washing buffer (0.01 M PBS containing 0.05% (w/v) Tween-20, pH 7.4). Finally, the constructed aptasensor was kept at 4 °C until use.

### Electrochemical analysis

Exosomes were added on the surface of GCE and incubated at 37 °C for 2 h. Then, the PD-L1 Apt.@PdCuB MNs nanotags were added and maintained at 37 °C for 2 h. After each step, excess unbound substances were washed out with PBS (10 mM, pH 7.4). The working GCE was immersed in PBS (10 mM, pH 7.4) containing H_2_O_2_, and the differential pulse voltammetry (DPV) was measured with an electrochemical workstation under the conditions of potential scanning voltage of 0.15 to − 0.65 V, amplitude of 70 mV, pulse width of 50 ms and pulse period of 200 ms.

### Clinical samples detection

Human serum samples were collected from healthy donors and NSCLC patients at the First Affiliated Hospital of Chongqing Medical University. All experiments on clinical samples were approved by the ethic committee of the First Affiliated Hospital of Chongqing Medical University. Clinical samples were centrifuged at 3000 × g for 20 min to remove large particles and cellular debris, and the supernatant was filtered through a 0.22 μm syringe filter and diluted with PBS (10 mM, pH 7.4) for the assessment of the aptasensor.

### Statistical analysis

Statistical analysis was performed using IBM SPSS Statistics 22.0 software (IBM Corp., Armonk, NY, USA). Group difference was assessed using Mann–Whitney test: ** *p* < 0.01.

## Results and discussion

### *Preparation and characterization of the exosomes, PdCuB MNs nanotags and Au@CuCl*_*2*_* NWs*

The exosomes were characterized by TEM and NTA. As shown in Additional file 1: Fig. S1A, the exosomes exhibited a typical cup-shaped morphology*.* Furthermore, the NTA results (Additional file 1: Fig. S1B) suggested a peak corresponding to a particle diameter of 103.3 nm with a concentration of 1.6 × 10^6^ particles/mL (diluted in a ratio of 1:1000).

**Fig. 1:**
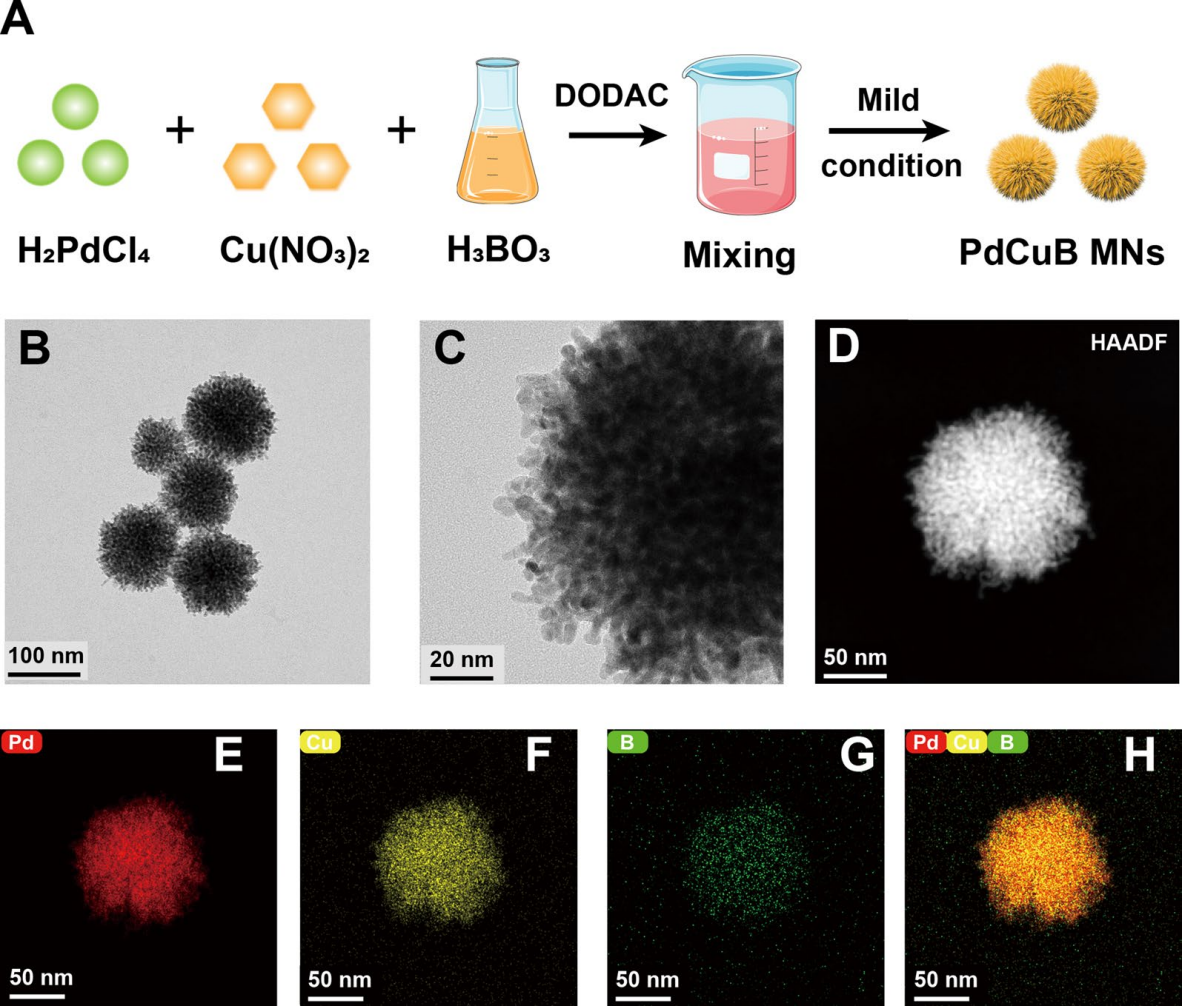
Synthesis and characterizations of PdCuB MNs. **A** The detailed synthesis process of PdCuB MSNs. **B** TEM image of PdCuB MNs. **C** HRTEM image of PdCuB MNs. **D** HAADF-STEM image of PdCuB MNs. **E**–**H** STEM-EDS elemental mapping of PdCuB MNs

Figure 1A depicts the facile synthesis of PdCuB MNs. TEM image clearly showed that PdCuB MNs were monodisperse and spherical with a diameter of about 112 nm (Fig. 1B). In addition, HRTEM and HAADF-STEM images demonstrated that PdCuB MNs had a microporous network structure (Fig. 1C, D). Specifically, Additional file 1: Fig. S2 revealed that the micropore size of PdCuB MNs was 1.46 nm and the backbone thickness was 3.17 nm. EDS elemental mapping showed the homogeneous distribution of the main components Pd, Cu and B throughout the microporous nanostructure (Fig. 1E–H). These results indicated that ternary PdCuB MNs nanotags were successfully synthesized.

The as-prepared Au@CuCl_2_ NWs exhibited a typical wire-like structure with an uneven length of 1717 ~ 4196 nm and a uniform width of ~ 114 nm (Additional file 1: Fig. S3A, B). EDS elemental analysis demonstrated the homogeneous distribution of Cu, Cl, and Au in the nanowires (Additional file 1: Fig. S4A–D). In addition, we performed AFM characterization on the surface of the constructed biosensor electrode. As shown in Additional file 1: Fig. S5, the results clearly demonstrated that compared to the bare electrode, significant nanowire adsorption was observed on the surface of the working electrode, which also confirmed the successful construction of proposed biosensor. Above results confirmed that Au@CuCl_2_ NWs were successfully prepared.

### Electrochemical behaviors of the nanomaterials and the designed sandwich aptasensor

To explore the peroxidase-like activity of PdCuB MNs, the steady-state kinetics of the oxidation of H_2_O_2_ was studied. The kinetics data were obtained by performing a series of experiments. To obtain the kinetic parameters, the data were fitted to Michaelis–Menten equation (Additional file 1: Fig. S6). Moreover, Michaelis–Menten constant (K_m_) and the maximal reaction velocity (V_max_) were calculated according to the reported literature [24, 25]. The results were shown in Additional file 1: Table S2, which evidenced that PdCuB MNs possessed lower K_m_ and higher V_max_ values compared with other nanoenzyme (PdPtCu nanosheets and PdAgB MNs), indicating superior catalytic activity toward H_2_O_2_.

In addition, to visually verify the H_2_O_2_ catalytic ability of Au@CuCl_2_ NWs, we also performed TMB colorimetric experiments to demonstrate whether the nanowires possess peroxidase-like activity. As shown in Additional file 1: Fig. S7, there was no significant color change of Au@CuCl_2_ NWs compared with the control PdCuB MNs, indicating that Au@CuCl_2_ NWs could not catalyze the decomposition of H_2_O_2_.

**Fig. 2:**
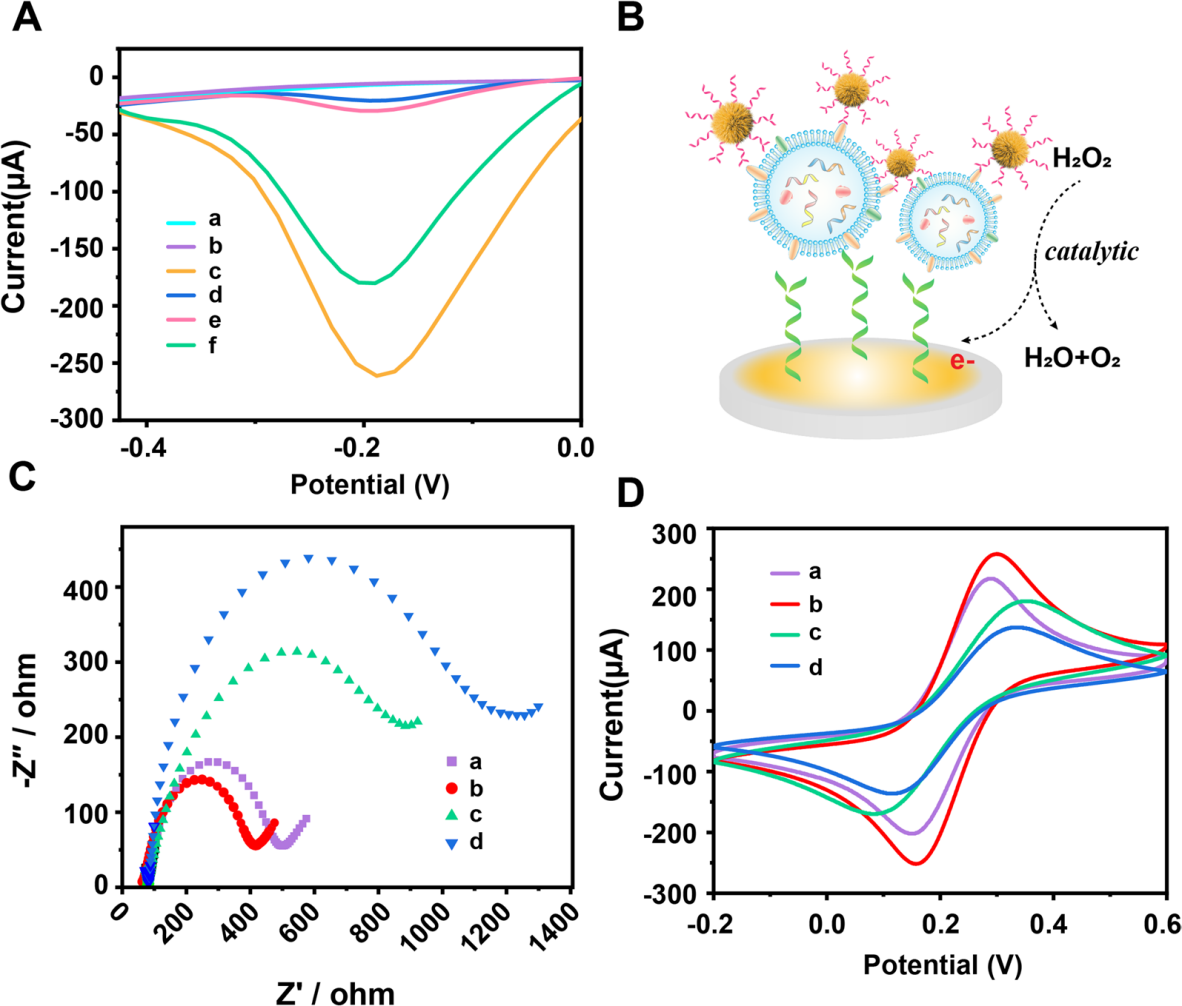
**A** DPV analysis of the designed aptasensor: bare GCE (a), Au@CuCl_2_ NW-modified GCE (b), PdCuB MNS-modified GCE (c), PdCuB MNs nanotag-based biosensor without target exosomes (d), PdCuB MNs without PD-L1 aptamer-based biosensor (e) and PdCuB MNs nanotag-based biosensor with target exosomes (f). DPV measurements were executed in 0.01 M PBS (pH 7.4) with 5 mM H_2_O_2_ in a potential range of 0.15 ~ − 0.65 V. **B** Signal transduction process of electrochemical exosome aptasensor. EIS **C** and CV **D** characterization of bare GCE (a), Au@CuCl_2_ NWs/GCE (b), CD63 Apt./Au@CuCl_2_ NWs/GCE (c), exosomes/CD63 Apt./Au@CuCl_2_ NWs/GCE (d). In the EIS measurements, the frequency range was 0.02 Hz ~ 100 kHz, the amplitude was 5 mV, and the CV measurements ranged from − 0.2 V to 0.6 V vs. Ag/AgCl reference at 0.15 V/s

To further validate the performance of PdCuB MNs toward the H_2_O_2_ electrochemical system, DPV measurements were conducted on an electrochemical workstation. A significantly increased DPV signal was observed on the PdCuB MNs-modified electrode (curve c), whereas no DPV response was observed in the bare GCE (curve a) and Au@CuCl_2_ NW-coated GCE (curve b) (Fig. 2A). The above results indicated that PdCuB MNs could be employed as nanotags to catalyze H_2_O_2_ for generating electrochemical signal, which was not affected by Au@CuCl_2_ NWs (Fig. 2B). We further investigated the thermal and long-term stability of PdCuB MNs in serum. As seen from Additional file 1: Fig. S8A, when the reaction temperature is gradually increased from 20 to 37 °C, the electrocatalytic activity of PdCuB MNs keeps constant, indicating that PdCuB MNs can maintain its activity in the working environment of the developed sensor. As shown in Additional file 1: Fig. S8B, PdCuB MNs can retain more than 95% of their original electrocatalytic activity after immersion in serum for more than 8 h. Therefore, we believe that PdCuB MNs have excellent electrocatalytic activity which is sufficient to meet the serum stability requirements for clinical assays.

The feasibility of the developed sandwich electrochemical aptasensor for PD-L1^+^ exosome detection was further investigated. The DPV signal of the PdCuB MNs nanotag-based sandwich aptasensor incubated with PD-L1^+^ exosome (curve f) was obviously larger than without exosomes and bare PdCuB MNs with exosomes (Fig. 2A, curve d and e), revealing the specific and efficient recognition of PD-L1 Apt.@PdCuB MNs nanotags and PD-L1^+^ exosome, as well as the well-maintained electrocatalytic activity facilitate the output electrochemical signals. These results demonstrated the fabricated sandwich electrochemical aptasensor were applicable to the detection of PD-L1^+^ exosome.

To validate the stepwise assembly process of the modified electrode, electrochemical impedance spectroscopy (EIS) and cyclic voltammetry (CV) were performed in 5 mM Fe(CN)_6_^3−/4−^ containing 0.1 M KCl. The electron transfer resistance (Ret) details about the fabricated aptasensor could be acquired from EIS by measuring a semicircle diameter in the Nyquist diagram. As shown in Fig. 2C, the Nyquist plot included a small impedance value for the bare GCE (curve a). Nevertheless, the diameter of the semicircle clearly decreased after coating Au@CuCl_2_ NWs on the electrode (curve b). The results revealed that Au@CuCl_2_ NWs could enhance electron transfer between GCE and electrolyte solution. This may be due to the presence of Cu atoms reduces the binding energy of Au 4f orbital electrons, and Cl additives is able to improve conductivities and carrier mobility [26, 27]. Moreover, the one-dimensional anisotropic structure of the nanowires and the large surface-to-volume ratio provide more electroactive sites [26, 28]. As expected, the impedance markedly increased with the CD63 Apt. tethered on the Au@CuCl_2_ NWs-modified GCE (curve c), owing to the hindered effect of Apt. on electron transport rate [29]. Subsequently, the impedance further increased after captured the exosomes (curve d) due to the electron inertia of the specific interacted CD63 Apt. and CD63 protein on the exosomes, which impeded the electron and mass transport of Fe(CN)_6_^3−/4−^ [4]. In addition, the REDOX current peaks were measured for confirming the stepwise variation of the modified electrode. As exhibited in Fig. 2D, the current of Au@CuCl_2_ NW-modified GCE (curve b) significantly increased compared to that of bare electrode (curve a) due to the acceleration of electron transfer and internal reduction of resistance by Au@CuCl_2_ NWs. The peak current of the electrode declined after modified with CD63 Apt., and further decreased upon the binding of CD63 Apt. and PD-L1^+^ exosomes, which are both due to the high resistivity of CD63 and exosomes as non-conductive biological components, thus affecting the electron transfer rate [29]. The sandwich aptasensor was well-fabricated and displayed a good response to the targeted PD-L1^+^ exosome.

Further, the stability of Au@CuCl_2_ adsorbed on the GCE was investigated by CV analysis. As shown in Additional file 1: Fig. S9, the height of the oxidation and reduction peak of Au@CuCl_2_ NW-modified GCEs decreased slightly with the extension of time at room temperature, which confirmed the stability of Au@CuCl_2_ nanowires meets the application requirements.

### Factors affecting sandwich aptasensing

**Fig. 3:**
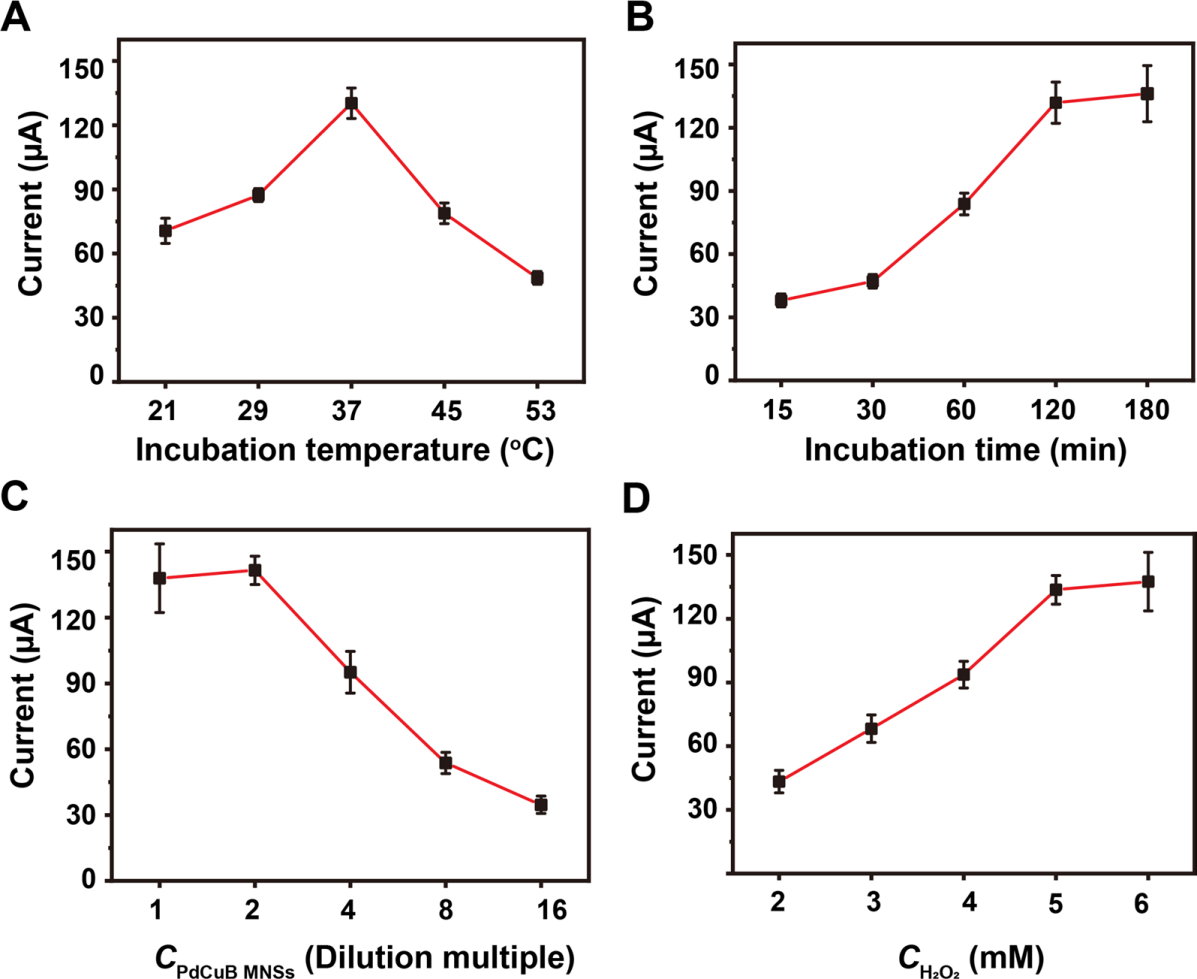
Optimization of **A** the incubation temperature of exosomes, **B** incubation time of exosomes, **C** dilution multiple of PdCuB MNs and **D** concentration of H_2_O_2_

To achieve the optimal analytical performance, a series of key experimental parameters were systematically investigated. The key factors for exosome viability, including incubation time and temperature were optimized firstly. The results indicated that the optimum incubation temperature and time are 37 °C and 120 min, respectively (Fig. 3A, B). Besides, the concentration of PdCuB MNs nanotags is closely connected to the electrocatalytic activity and manufacturing cost. As shown in Fig. 3C, the aptasensor exhibited a maximum DPV value at two-fold of PdCuB MNs nanotags, which was utilized as the optimum concentration of PdCuB MNs nanotags. Furthermore, 5 mM H_2_O_2_ in working solution was selected due to the maximum current signal (Fig. 3D).

### Analytical performance of the developed aptasensor

**Fig. 4:**
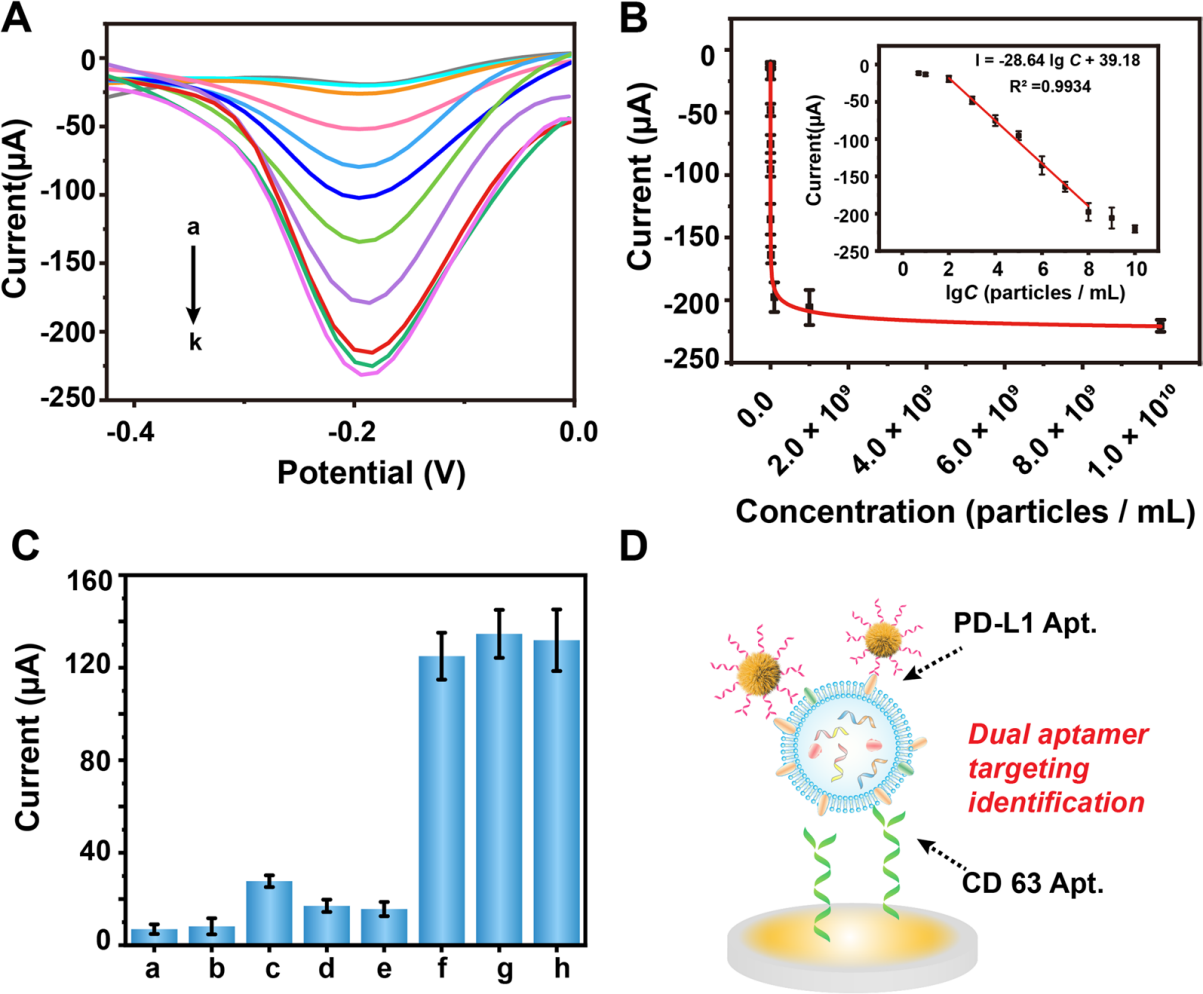
**A** DPV signals of the sandwich electrochemical aptasensor with different concentrations of PD-L1^+^ exosomes (a–k: 5, 1 × 10^1^, 1 × 10^2^, 1 × 10^3^, 1 × 10^4^, 1 × 10^5^, 1 × 10^6^, 1 × 10^7^, 1 × 10^8^, 1 × 10^9^, 1 × 10^10^ particles/mL). **B** DPV signals of the aptasensor incubated with different concentrations of PD-L1^+^ exosomes. Inset: calibration plot of the DPV value vs. the logarithm of the PD-L1^+^ exosome concentration. **C** DPV responses with different targets: 50 ng/mL BSA (a), 50 ng/mL ALB (b), 50 ng/mL PD-L1 (c), 1 × 10^7^ particles/mL BEAS-2B derived exosomes (d), 1 × 10^7^ particles/mL L-02 derived exosomes (e), 1 × 10^6^ particles/mL A375 derived exosomes (f), a mixture (1 × 10^6^ particles/mL PD-L1^+^ exosomes + 1 × 10^7^ particles/mL BEAS-2B-derived exosomes + 50 ng/mL BSA) (g) and 1 × 10^6^ particles/mL PD-L1^+^ exosomes (h). Error bars: SD, n = 3. **D** Diagram of exosome recognition model

With the optimal reaction conditions, the exosomes derived from A549 cells was examined via the developed aptasensor. Figure 4A displayed the DPV current values for detecting different concentrations of exosomes ranging from 5 particles/mL (curve a) to 1 × 10^10^ particles/mL (curve k). Additionally, the plot of DPV intensity against exosome concentration was presented in Fig. 4B. In addition, the current (*I*) decreased with the logarithm of exosome concentration (*C*, particles/mL) with a linear regression equation *I* = − 28.64 lg *C* + 39.18 in a range from 1 × 10^2^ to 1 × 10^8^ particles/mL with a determination coefficient of 0.9934 and a limit of detection of 36 particles/mL (*S/N* = 3), which are comparable with most previously reported high-performance exosome biosensors [30–33] (Fig. 4B, inset; Additional file 1: Table S3). Such excellent performance of the developed aptasensor could be attributed to the efficient catalytic activity of PdCuB MNs nanotags towards H_2_O_2_ and the high electrical conductivity of Au@CuCl_2_ NWs.

Selectivity and anti-interference of the fabricated aptasensor were investigated. Bovine serum albumin (BSA), albumin (ALB), PD-L1 protein, human bronchial epithelial cell (BEAS-2B) and human hepatocyte cell (L-02)-derived exosomes were used as interference agents, and human melanoma cells (A375) and A549 cell-derived exosomes were used as positive controls. As displayed in Fig. 4C, these five interferents made negligible response to the developed sandwich aptasensor and did not produce distinguishable effect on the detection of PD-L1^+^ exosome. The above results revealed the good selectivity and anti-interference capacity of the developed aptasensor for PD-L1^+^ exosome, which may result from the high-affinity recognition of the dual-Apt. and the delicate nonspecific response of nanomaterials (Fig. 4D) [10, 34].

We have detailed evaluated the stability and repeatability of the proposed electrochemical biosensor. The electrochemical assay signals for the same exosome concentration were evaluated for 8 consecutive days and 10 repetitions of different concentration gradients. According to Additional file 1: Fig. S10A, the DPV signal decreased gradually and retained 91.38% of the initial current after being stored at 4 °C for 8 days. Correspondingly, Additional file 1: Fig. S10B shows that there was little difference between the results within a measurement circle (Relative standard deviation (RSD) = 3.9%, 2.6%, 3.5%). It demonstrated the excellent detection stability and repeatability of the biosensor.

### Clinical sample analysis

To assess the clinical analysis performance of the designed aptasensor, the developed aptasensor was applied to the quantitative analysis of target exosomes spiked in human serum samples. Quantitative recoveries were obtained ranging from 96.61 to 100.99% with RSDs ranging from 2.09 to 4.81% (n = 3) (Table 1). Additionally, clinical samples were determined to further verify the clinical utility of the aptasensor.

**Table 1:** Determination of A549 derived exosomes spiked in PD-L1^+^ exosome-negative serum samples by using the developed electrochemical aptasensor

Sample no	Added (particles/mL)	DPV (μA)	Found (particles/mL)	Recovery (%)	RSD (%)
1	1000	− 46.6	989	98.90	2.09
2	100000	− 104.1	100990	100.99	4.81
3	10000000	− 160.9	9660608	96.61	2.96

**Fig. 5:**
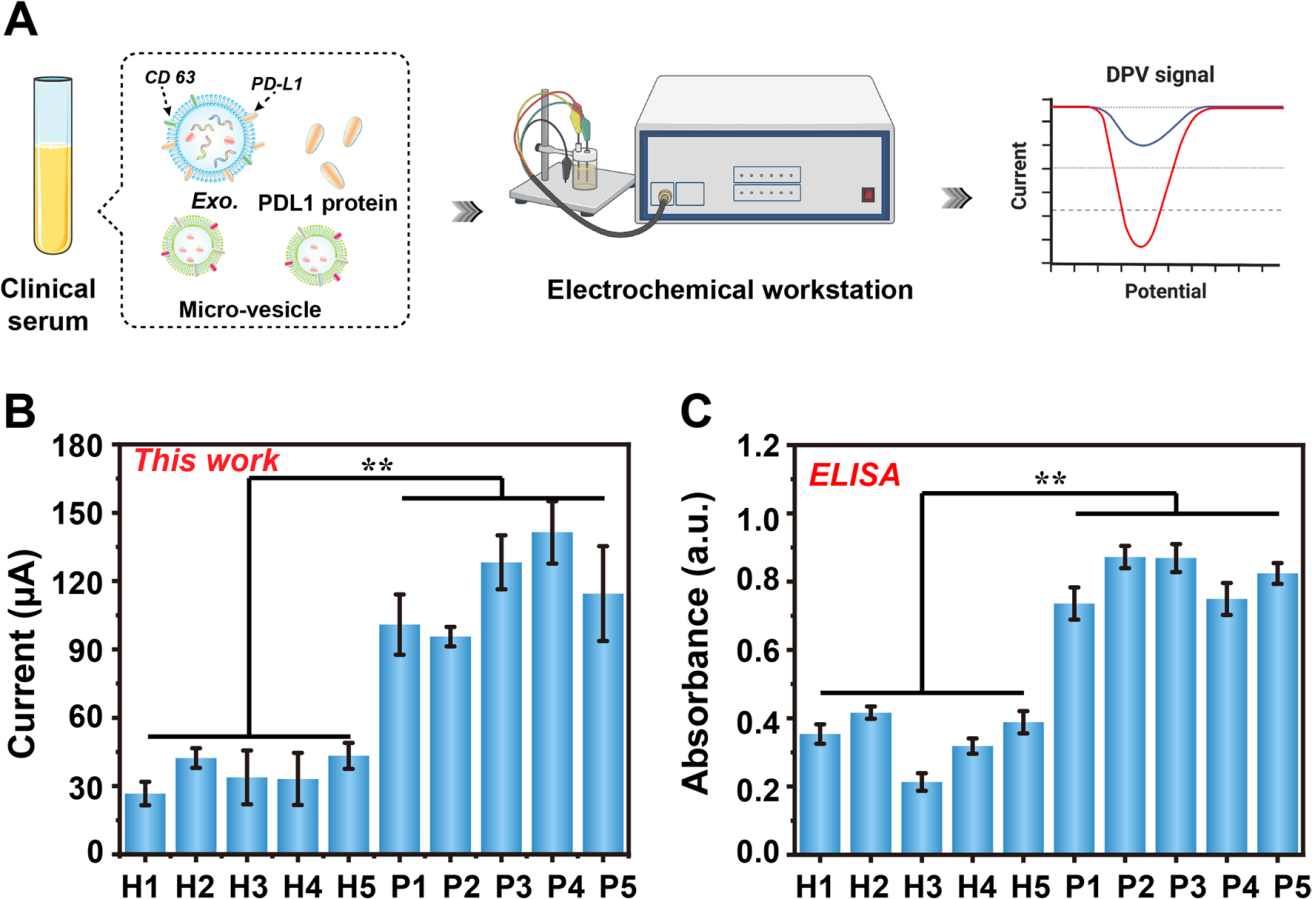
Clinical samples analysis by using the developed electrochemical aptasensor (**, *p* < 0.01)

To further demonstrate the credibility of the fabricated biosensor, we collected 10 serums from healthy volunteers and NSCLC patients and performed PD-L1^+^ exosome detection by ELISA and the proposed biosensor, respectively. As shown in Fig. 5, the test results of the biosensor could clearly distinguish the PD-L1^+^ exosome expression levels of healthy volunteers and NSCLC patients, and maintained favorable consistency with the ELISA assay data. Based on the linear regression equation, we precisely analyzed the amount of PD-L1^+^ exosomes in clinical samples, with 200–756 and 50749–2019145 particles/mL in the serum of healthy volunteers as well as NSCLC patients, respectively, which is consistent with the previous clinical studies reported [35]. The above outcomes demonstrated that the biosensor constructed in this study possessed promising detection performance and potential clinical application.

## Conclusion

In summary, we successfully developed a novel sandwich electrochemical aptasensor for ultrasensitive and highly specific analysis of PD-L1^+^ exosomes. The PdCuB MNs served as a powerful nanotag, exhibited significant peroxide-like activity, and combined with conductive Au@CuCl_2_ NWs to impart a high-intensity electrochemical signal, making the aptasensor meet the low abundance exosome detection requirement. In addition, the highly specific and excellent anti-interference for the determination of PD-L1^+^ exosome in complex matrices was achieved by employing dual Apt. targeting recognition. The electrochemical aptasensor was successfully applied to exosome detection in clinical samples and was consistent with the clinical results. The developed electrochemical aptasensor provides a reliable technology for the early diagnosis of NSCLC and allows for broad application expansion through Apt. sequence variation.

## Supplementary Information

**Additional file 1: Table S1**. Oligonucleotides used in the present work. **Fig. S1** Characterizations of exosomes derived from A549 cells. **Fig. S2** High-resolution TEM images of PdCuB MNs. **Fig. S3** Structural characterizations of the Au@CuCl_2_ NWs. **Fig. S4** STEM-EDS elemental mapping of Au@CuCl_2_ NW. **Fig. S5** AFM characterization of Au@CuCl_2_ NWs on the surface of the constructed biosensor electrodes. **Fig. S6** Kinetic assay for the peroxidase-like activity of nanozymes. **Table S2**. Kinetic parameters for the peroxidase-like activity of nanozymes. **Fig. S7** TMB chromogenic reaction images. **Fig. S8** Stability of PdCuB MNs in serum. **Fig. S9** The stability of Au@CuCl_2_ NWs for adsorbed on the GCE surface. **Fig. S10** The long-term stability and repeatability of electrochemical biosensor for PD-L1^+^ exosomes. **Table S3**. Comparison of the fabricated aptasensor with other developed biosensors for PD-L1^+^ exosome detection.

## Data Availability

All data generated or analyzed during this study are included in this article and the Additional Information. The additional file is available.
