# Supplementary material for: Microporous PdCuB nanotag-based electrochemical aptasensor with Au@CuCl2 nanowires interface for ultrasensitive detection of PD-L1-positive exosomes in the serum of lung cancer patients
Source: J Nanobiotechnology. 2023 Mar 11;21:86. doi: 10.1186/s12951-023-01845-y (PMC10008610; doi:10.1186/s12951-023-01845-y)
Supplement: Supplementary file 1 — Additional file 1: Table S1. Oligonucleotides used in the present work. Fig. S1 Characterizations of exosomes derived from A549 cells. Fig. S2 High-resolution TEM images of PdCuB MNs. Fig. S3 Structural characterizations of the Au@CuCl2 NWs. Fig. S4 STEM-EDS elemental mapping of Au@CuCl2 NW. Fig. S5 AFM characterization of Au@CuCl2 NWs on the surface of the constructed biosensor electrodes. Fig. S6 Kinetic assay for the peroxidase-like activity of nanozymes. Table S2. Kinetic parameters for the peroxidase-like activity of nanozymes. Fig. S7 TMB chromogenic reaction images. Fig. S8 Stability of PdCuB MNs in serum. Fig. S9 The stability of Au@CuCl2 NWs for adsorbed on the GCE surface. Fig. S10 The long-term stability and repeatability of electrochemical biosensor for PD-L1+ exosomes. Table S3. Comparison of the fabricated aptasensor with other developed biosensors for PD-L1+ exosome detection. [file 12951_2023_1845_MOESM1_ESM.docx]

**Additional file 1**

**Microporous PdCuB nanotag and Au@CuCl_2_ nanowire-based ultrasensitive electrochemical aptasensor for early diagnosis of non-small cell lung cancer**

Luyue Chang^a^, Haiping Wu^c^, Rui Chen^c^, Xiaoqing Sun^b^, Yi Yang^b^, Changwu Huang^b^, Shijia Ding^c^, Changjin Liu^a, b, *^, Wei Cheng^a, *^

*^a^ The Center for Clinical Molecular Medical Detection, The First Affiliated Hospital of Chongqing Medical University, Chongqing 400016, China*

*^b^ Department of Laboratory Medicine, The Fifth People's Hospital of Chongqing, Chongqing, 400062, China*

*^c^ Key Laboratory of Clinical Laboratory Diagnostics (Ministry of Education), College of Laboratory Medicine, Chongqing Medical University, Chongqing, 400016, China*

* Corresponding author: E-mail address: [chengwei@hospital.cqmu.edu.cn](mailto:chengwei@hospital.cqmu.edu.cn) (CW) and [liuxin_27@foxmail.com](mailto:liuxin_27@foxmail.com) (LC).

**Table S1**. Oligonucleotides used in the present work

| Name | *Sequence (5'-3') |
| --- | --- |
| CD63 aptamer | CACCCCACCTCGCTCCCGTGACACTAATGCTA**TTTT**-(CH_2_)_6_-SH |
| PD-L1 aptamer | SH-(CH_2_)_6_-**TTTT**TACAGGTTCTGGGGGGTGGGTGGGGAAC CTGTT |


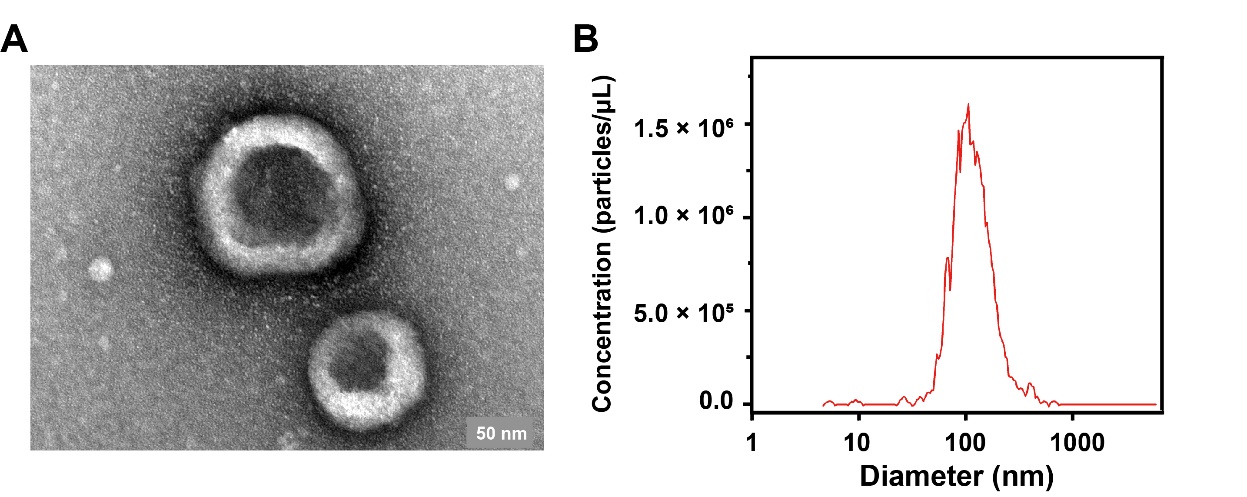


**Fig. S1** Characterizations of exosomes derived from A549 cells. A) TEM image of the isolated exosomes (scale bar: 50 nm). B) NTA of the isolated exosomes.


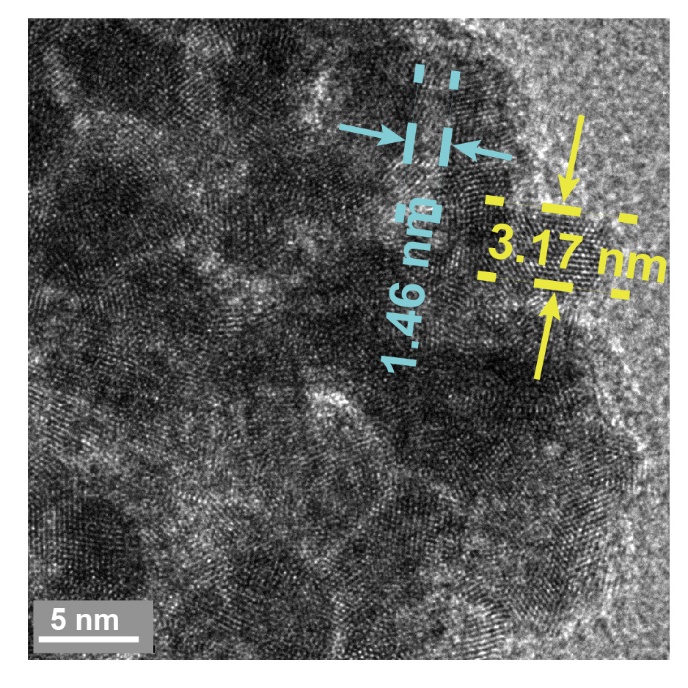


**Fig. S2** High-resolution TEM images of PdCuB MNs.


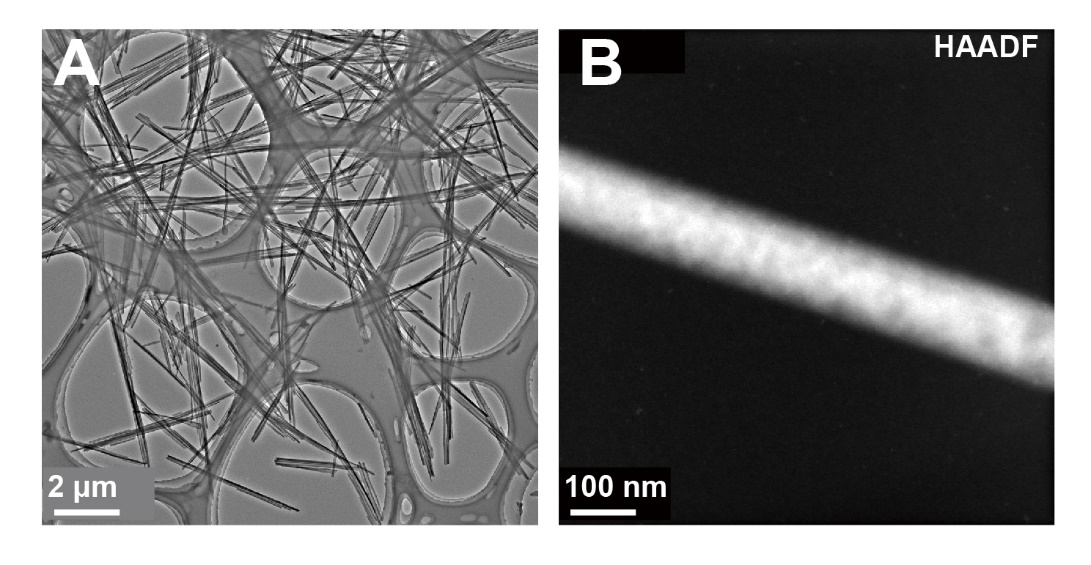


**Fig. S3** Structural characterizations of the Au@CuCl_2_ NWs. A) TEM image of Au@CuCl_2_ NWs. B) HAADF-STEM image of Au@CuCl_2_ NW.


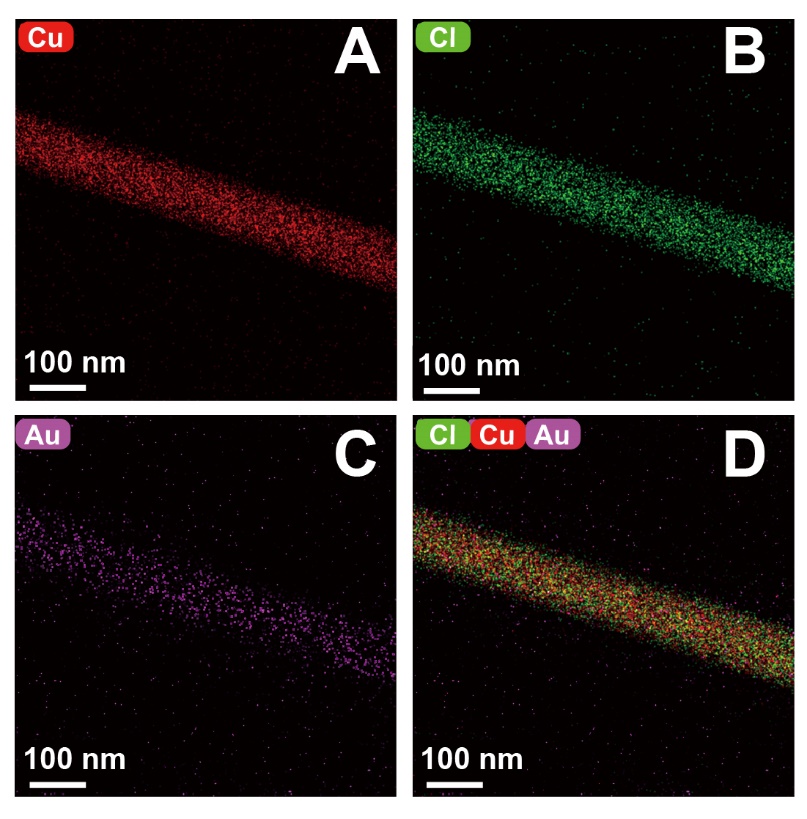


**Fig. S4** STEM-EDS elemental mapping of Au@CuCl_2_ NW.

**
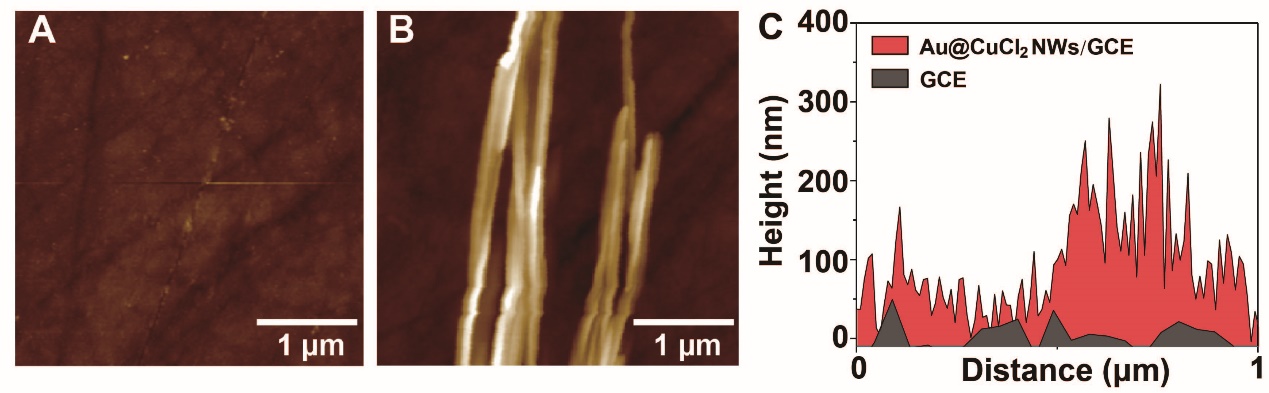
**

**Fig. S5.** AFM characterization of Au@CuCl_2_ NWs on the surface of the constructed biosensor electrodes. A) Bare GCE. B) Au@CuCl2 NWs/GCE, and C) The extracted profiles of both samples.


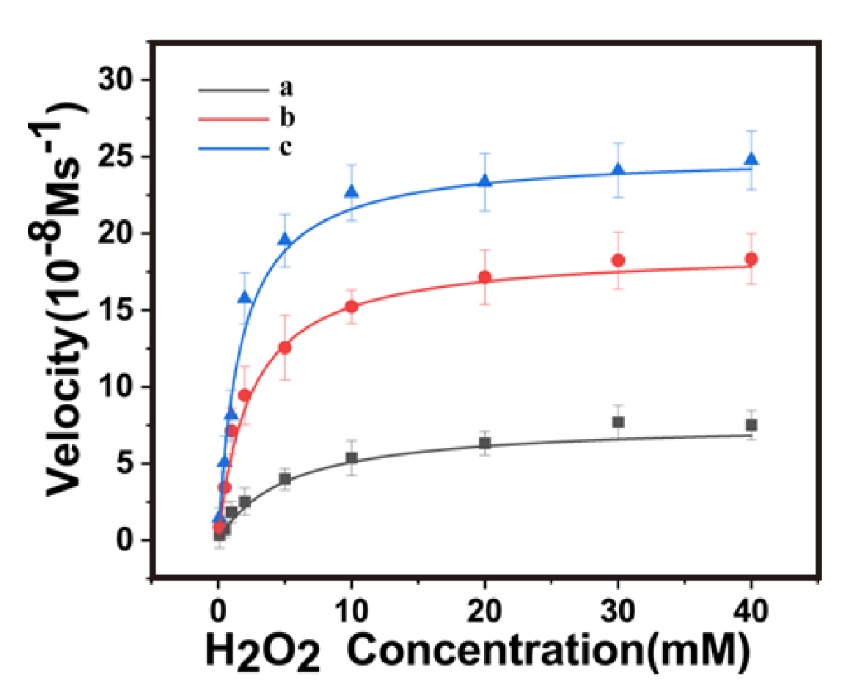


**Fig. S6**. Kinetic assay for the peroxidase-like activity of PdAgB MNs (a), PdPtCu nanosheets (b), and PdCuB MNs (c) with H_2_O_2_ as substrate.

**Table S2** Kinetic parameters for the peroxidase-like activity of PdCuB MNs, PdPtCu nanosheets and PdAgB MNs with H_2_O_2_ as substrate.

| Nanoenzymes | K_m_ (mM) | V_max_ (10^−8^ Ms^−1^) |
| --- | --- | --- |
| PdCuB MNs | 1.68 | 25.21 |
| PdPtCu nanosheets | 2.43 | 18.91 |
| PdAgB MNs | 5.15 | 7.71 |

**
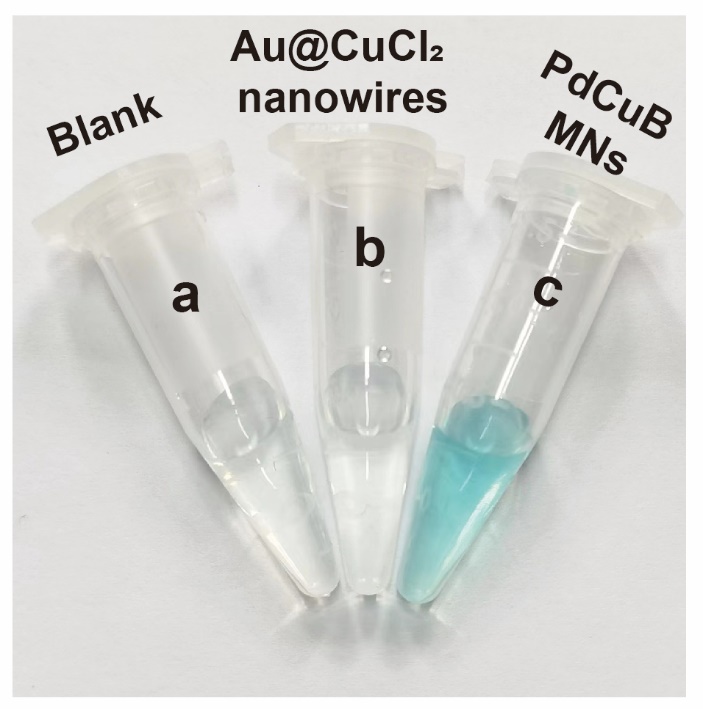
**

**Fig.** **S7.** TMB chromogenic reaction images: (a) Blank; (b) Au@CuCl_2_ nanowires; (c) PdCuB MNs.

**
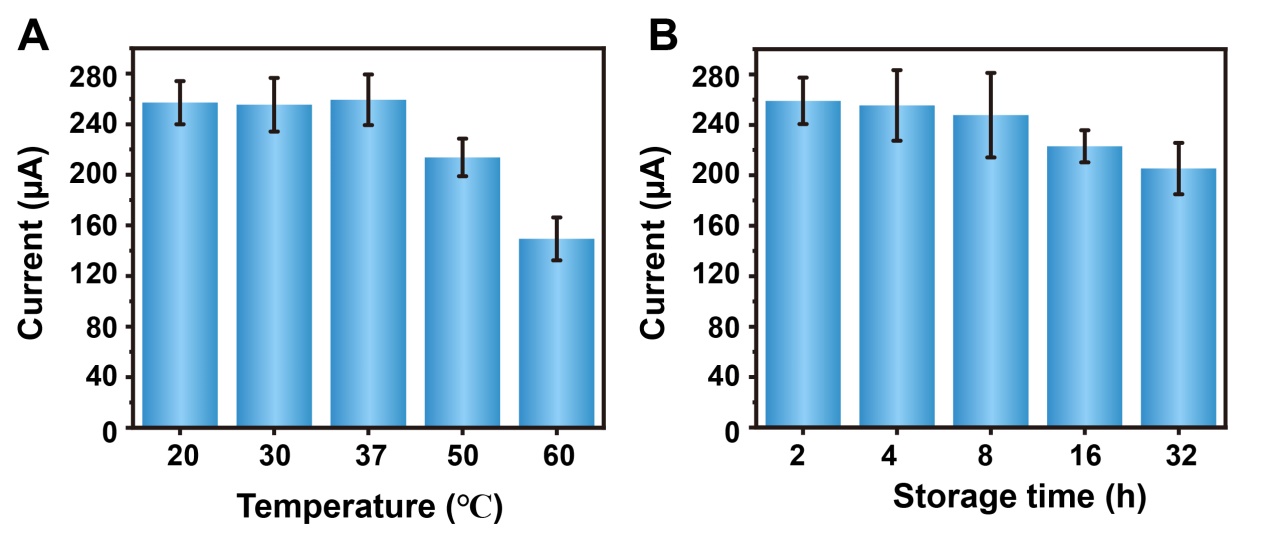
**

**Fig.** **S8.** (A) Thermal stability of PdCuB MNs in serum under different temperatures. (B) Long-term stability of PdCuB MNs in serum.

**
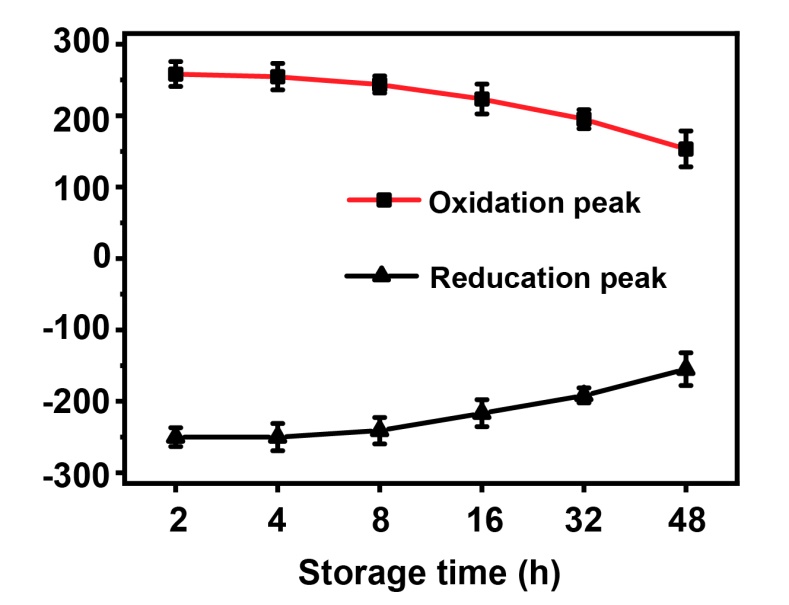
**

**Fig.** **S9**. The stability of Au@CuCl_2_ for adsorbed on the GCE surface. Error bars: SD, n=3.

**
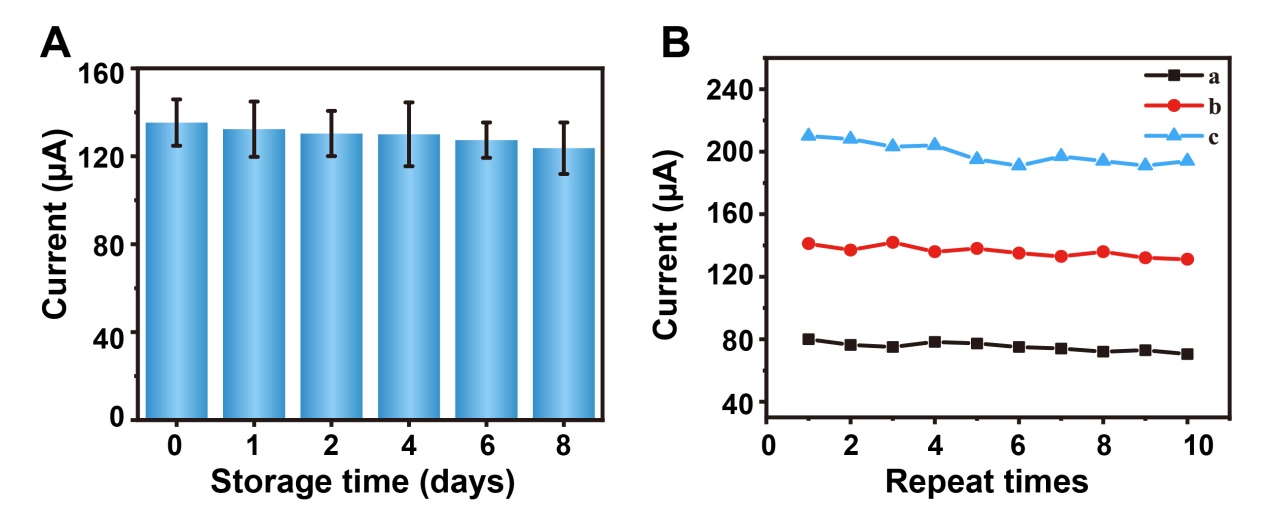
Fig.** **S10.** (A) The long-term stability of electrochemical biosensor for PD-L1^+^ exosome (1 × 10^6^ particles/mL) detection. Error bars: SD, n=3. (B) The repeatability of the electrochemical biosensor. (a–c: 1 × 10^4^, 1 × 10^6^, 1 × 10^8^ particles/mL PD-L1^+^ exosomes).

**Table S3.** Comparison of the fabricated aptasensor with other developed biosensors for PD-L1^+^ exosome detection.

| Reporter Molecule | Substrate | Method | Linear Range | LOD | Ref. |
| --- | --- | --- | --- | --- | --- |
| None | GO/AuNTs | EIS | 1 × 10^2^ – 1 × 10^6^ particles/mL | 76 particles/mL | [1] |
| Au@Ag@MBA | Fe3O4@TiO_2_ | SERS | 5 × 10^3^ – 2 × 10^5^ particles/mL | 1 particles/μL | [2] |
| MB | COF-367 NSs | SWV | 1.2 × 10^2^ – 1.2 × 10^7^ particles/μL | 38 particles/μL | [3] |
| None | Graphene | SPR | 1 × 10^4^ – 1 × 10^8^ particles/mL | 20 particles/mL | [4] |
| PdCuB MNSs | **Au@CuCl_2_ NWs** | **DPV** | **1 × 10^2^ – 1 × 10^8^ particles/mL** | **36 particles/mL** | **This work** |

**Notes**: EIS: Electrochemical impedance spectroscopy. SERS: Surface-enhanced Raman scattering. SWV: Square wave voltammetry. SPR: Surface plasmon resonance. DPV: Differential pulse voltammetry.

**References**

1. Mao Z, Wang Y, Chen Q, Zhu Z, Koh K, Chen X, et al. Multifunctional Peptides Modified Conductive Nano-Network Based on GO and Gold Nano Triangular: Sensitive Detection of PD-L1 Exosomes in Serum. J Electrochem Soc. 2022;169:076505.

2. Pang Y, Shi J, Yang X, Wang C, Sun Z, Xiao R. Personalized detection of circling exosomal PD-L1 based on Fe_3_O_4_@TiO_2_ isolation and SERS immunoassay. Biosens Bioelectron. 2020;148:111800.

3. Liu F, Yang Y, Wan X, Gao H, Wang Y, Lu J, et al. Space-Confinment-Enhanced Fluorescence Detection of DNA on Hydrogel Particles Array. ACS Nano. 2022;16:6266-73.

4. Mao Z, Zhao J, Chen J, Hu X, Koh K, Chen H. A simple and direct SPR platform combining three-in-one multifunctional peptides for ultra-sensitive detection of PD-L1 exosomes. Sens Actuator B-Chem. 2021;346:130496.
